# Nutritional Care Practices in Geriatric Rehabilitation Facilities across Europe: A Cross-Sectional Study

**DOI:** 10.3390/jcm12082918

**Published:** 2023-04-17

**Authors:** Irma H. J. Everink, Stefan Grund, Petra Benzinger, Anne de Vries, Adam L. Gordon, Janneke P. van Wijngaarden, Jürgen M. Bauer, Jos M. G. A. Schols

**Affiliations:** 1 Department of Health Services Research, Care and Public Health Research Institute (CAPHRI), Maastricht University, 6200 MD Maastricht, The Netherlands; 2 Center for Geriatric Medicine, Agaplesion Bethanien Hospital Heidelberg, Geriatric Center at the Heidelberg University, 69126 Heidelberg, Germany; 3 Institute for Health and Generations, University of Applied Sciences Kempten, 87435 Kempten, Germany; 4 Danone Trading Medical B.V., 2132 LS Hoofddorp, The Netherlands; 5 Academic Unit of Injury, Recovery and Inflammation Sciences (IRIS), School of Medicine, University of Nottingham, Nottingham NG7 2UH, UK; 6 NIHR Applied Research Collaboration-East Midlands (ARC-EM), Nottingham DE22 3NE, UK; 7 Danone Nutricia Research, 3584 CT Utrecht, The Netherlands; 8 Department of Family Medicine and Care and Public Health Research Institute (CAPHRI), Maastricht University, 6200 MD Maastricht, The Netherlands

**Keywords:** malnutrition, sarcopenia, frailty, geriatric rehabilitation, post-acute care, screening, treatment, EUGMS

## Abstract

Many patients in geriatric rehabilitation (GR) are physically frail at the time of admission and suffer from malnutrition and sarcopenia, which may worsen rehabilitation outcomes. This study aims to obtain insight into the current nutritional care practices in GR facilities across Europe. Methods: In this cross-sectional study, a questionnaire focused on nutritional care practices in GR was distributed across experts in EUGMS member countries. Data were analyzed by using descriptive statistics. Results: In total, 109 respondents working in 25 European countries participated, and the results showed that not all GR patients were screened and treated for malnutrition, and not all participants used (inter)national guidelines when performing nutritional care. The results also showed variations across European geographical areas related to screening and treatment of malnutrition, sarcopenia, and frailty. Even though the participants underlined the importance of dedicating time to nutritional care, they experienced barriers in its implementation, which were mostly due to a lack of resources. Conclusion: As malnutrition, sarcopenia, and frailty are often present in patients admitted to GR, in addition to being interrelated, it is recommended to develop an integrated approach to screening and treatment of all three clinical problems.

## 1. Introduction

Geriatric rehabilitation (GR) is defined as a multidimensional approach to diagnostic and therapeutic interventions, the purpose of which is to optimize functional capacity, promote activity, and preserve functional reserve and social participation in older patients with disabling impairments. GR is recommended for patients affected by multimorbidity and geriatric syndromes who experience an acute event or show functional decline due to their long-term conditions and who have the potential to improve their functional outcome [1]. Most patients are admitted to GR after a hospital stay. Many GR patients are physically frail at the time of admission to GR and are affected by malnutrition and sarcopenia. These three clinical problems may worsen rehabilitation outcomes and contribute to subsequent disability or functional decline [2,3,4,5,6].

Malnutrition is defined as “a state resulting from lack of intake or uptake of nutrition that leads to altered body composition (decreased fat free mass) and loss of body cell mass (weight loss) leading to diminished physical and mental function, and impaired clinical outcome from disease” [7]. Malnutrition and the risk of malnutrition are very common in older hospitalized patients. The global prevalence depends on the diagnostic instrument used and the hospital population studied, and it varies from 59 to 98% at admission and from 30 to 88% at discharge [8,9]. There is also a high prevalence of malnutrition risk (47%) and malnutrition (13–43%) upon admission to GR [2,10]. Many nutritionally compromised patients do not consider themselves to be malnourished, and many do not seek or receive any form of nutritional attention or treatment [10].

Frailty is a geriatric syndrome with determinants related to rapid muscle fatigue. A generally accepted definition proposed by Fried et al. (2001) defines frailty as a clinical syndrome in which three or more of the following criteria are present: unintentional weight loss, self-reported exhaustion, weakness (low grip strength), slow walking speed, and low physical activity [11]. There is a relationship between muscle fatigue and the core elements of the cycle of frailty, as proposed by Fried et al., including sarcopenia and weight loss [12].

Sarcopenia is a muscle disease (muscle failure) characterized by a decrease in muscle strength and associated with an increased likelihood of adverse outcomes, such as falls, fractures, disability, and mortality [13,14,15,16,17]. Sarcopenia is highly prevalent upon hospital admission (35–37%) [18,19,20,21] and even increases in prevalence during hospital stays as a consequence of immobility and inactivity [19]. About 56% of patients discharged from the hospital to GR are sarcopenic [22]. Sarcopenia is multifactorial, with contributors including inflammatory processes associated with aging [23], acute illnesses, immobility during hospital stays [19,24], and malnutrition [19,20,21,25]. Despite the high prevalence of sarcopenia, as with malnutrition, there is evidence that it is underdiagnosed [26].

Malnutrition plays an important role in frailty and sarcopenia, which are highly prevalent in patients admitted for GR. This makes it very relevant to explore nutritional care practices in GR, all the more because there is little evidence available about the current nutritional care practices in geriatric rehabilitation facilities across Europe.

A survey by the European Geriatrics Medicine Society (EUGMS) found that in 80% of the participating countries, healthcare professionals indicated screening for malnutrition [27]. It remains unclear, however, what happens after screening; for example, it is unclear if nutritional intervention is initiated, as are the type and duration of nutritional therapy/treatment and whether and how patients are monitored during follow-up. In addition, the extent to which professionals screen for and treat sarcopenia and frailty is unknown. Therefore, gaining further insights into the clinical practice of nutrition in GR and differences in clinical practice between countries or regions within Europe could identify areas for quality improvement. The aim of this study was to provide an overview of the current nutritional care practices in GR across Europe.

## 2. Materials and Methods

### 2.1. Design

This is a descriptive cross-sectional study in which an online survey was designed and distributed to collect information on the nutritional care practices in geriatric rehabilitation across European countries that were members of the EUGMS in 2020.

### 2.2. Respondents

Respondents were eligible to participate if they were responsible for or knowledgeable about nutritional care in geriatric rehabilitation settings in their country.

### 2.3. Survey

The first draft of the online survey was developed by the authors S.G., A.d.V., and J.P.v.W, who are three experts in the field of nutritional care in GR. The content of the survey was partly based on nutritional care phases (e.g., screening/assessment and therapy) and interventions performed during these phases, as described by international guidelines [28]. The survey was piloted by the other authors, and based on their feedback, the questionnaire was finalized.

In the first part of the survey, the background characteristics of respondents were inquired about, including questions related to occupation, the country in which they worked, and their years of work experience in geriatric rehabilitation. Thereafter, the content-related questions were structured into five categories relating to the nutritional care practices in GR. The first category focused on the organization of nutritional care in the organization (e.g., is there someone/a team responsible for nutritional care, are patients screened for [risk of] malnutrition, and do patients receive targeted treatment for malnutrition?). The second part focused on screening and treatment of sarcopenia and frailty. The third part covered barriers and facilitators for performing nutritional interventions in the GR organization, and the fourth focused on healthcare practitioners’ expected impact of nutritional interventions on various clinical outcomes within the GR context. The last category of the survey focused on respondents’ thoughts about GR patients’ adherence to different nutritional treatments. This manuscript reports only the results provided for categories 1–3, in consensus with the aim of the study. The results for categories 4 and 5 will be described in a separate article. The questionnaire was developed in the online Google Forms tool. The full questionnaire is provided in the Supplementary Material.

### 2.4. Recruitment and Data Collection

With help of the European Geriatric Medicine Society (EuGMS), the survey was sent out in two waves: in July 2020 to all individual members (n = 44) of the EuGMS Special Interest Group (SIG) on Geriatric Rehabilitation and in April 2021 to all members of the EuGMS network. A delay was experienced in answering the survey due to the COVID-19 pandemic. The official members of the EuGMS-SIG were asked to distribute the survey to their colleagues in geriatric rehabilitation according to the snowballing technique. The EuGMS network includes medical and allied health experts (e.g., physiotherapists, occupational therapists) in the field of geriatric medicine from all EUGMS 38 member countries. The experts were people working in the field of geriatric medicine, as well as researchers.

### 2.5. Data Analysis

All data retrieved from Google Forms were exported to Microsoft Excel and subsequently manually imported into IBM SPSS Statistics, version 28 (IBM Corp., Armonk, NY, USA). The data were analyzed by using descriptive statistics. Because of the exploratory character of this study and the fact that it was not possible to obtain a representative sample from each country, no statistical analyses of the differences per country/region were performed.

## 3. Results

The survey was completed by 109 respondents working in 25 different countries across Europe (including Russia and Turkey). The countries that had the most respondents filling in the questionnaire were Spain (n = 19) and the Netherlands (n = 15). For this study, the respondents were grouped into four geographical categories: (1) Northern European/Nordic countries, including Sweden, Finland, Norway, Iceland, and Denmark (n = 17); (2) Central European/continental countries, including Ireland, the UK, the Netherlands, Belgium, Germany, France, Austria, and Switzerland (n = 43); (3) Southern European/Mediterranean countries, including Spain, Portugal, Malta, Italy, Turkey, and Greece (n = 42); (4) Eastern European countries, including Latvia, Russia, Belarus, Estonia, Hungary, and Slovenia (n = 7).

There was a large variation between the size and location where geriatric rehabilitation was provided. The number of beds ranged from 6 to 220, and the location where rehabilitation was provided varied from (academic) hospital wards to nursing homes, private clinics, and specific (geriatric) rehabilitation centers. Table 1 shows the background characteristics of the respondents. This table shows that the majority of the respondents were geriatricians (50%), followed by dieticians (37%). There was a large variety in work experience: The largest group (33%) had more than 20 years of working experience, followed by 0–5 years of working experience (19%). Some differences in working experience between the four geographical areas could be distinguished: Respondents in Eastern countries seemed to have less working experience, with the largest group consisting of respondents who had a working experience of between 0 and 5 years (43%), whilst the largest group in the Central European/continental countries had more than 20 years of working experience. Only 39% of the total sample population followed specific nutritional education related to rehabilitation or geriatric rehabilitation.

### 3.1. Dedicated Nutritional Care

**Figure 1:**
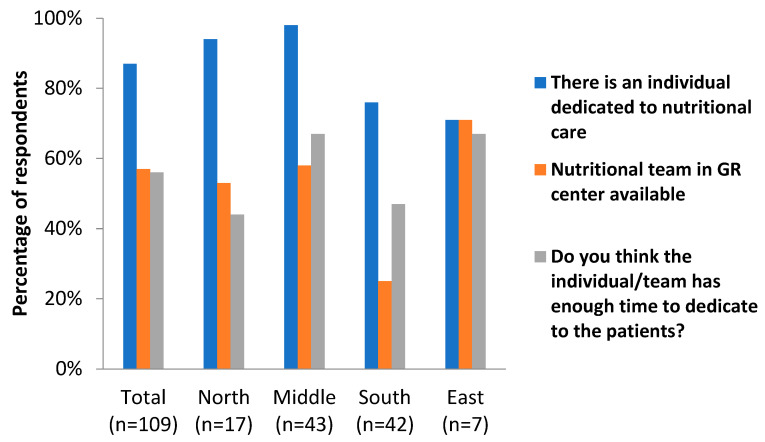
Dedicated nutritional care in GR centers in different geographical areas of Europe.

The first part of the survey focused on the presence or absence of dedicated nutritional care in geriatric rehabilitation centers (Table 2 and Figure 1). In 87% of the centers, there was at least one individual dedicated to nutritional care available. In the Nordic and Central European/continental countries, these percentages were above 90%, while these were around 70% in the Southern/Mediterranean and Eastern countries. In most of the centers, the individual dedicated to nutritional care was a dietician (67%), followed by a nutritionist (19%). Again, there seemed to be some differences between the geographical areas, as the dietician-led care was less prevalent in Southern and Eastern countries (41% and 29%, respectively) than in Northern and Central European countries (94% and 88%, respectively). Fifty-seven percent of the respondents indicated that there was a nutritional team in the GR facility. Members of this team were mostly dieticians, speech therapists, occupational therapists, and ‘other’ (physicians, nurses, and pharmacists were among the most frequently mentioned ‘other’ members). A little over half (56%) of the respondents indicated that the individual or team dedicated to nutritional care had enough time to execute their tasks for the patients. These percentages seemed higher for the Central European/continental and Eastern countries (67%) compared to the Northern European/Nordic and Southern European/Mediterranean countries (44 and 47%, respectively).

### 3.2. Reasons for the Lack of Time Dedicated to Nutritional Care

Respondents gave several reasons as to why the individual/team did not have enough time for nutritional care. No clear distinctions were seen among the answers given in the different regions, and the most common constraints were staff shortages; these were either because there was an insufficient full-time equivalent of staff or because the case load was too large to see all patients. In addition, ‘economic reasons’ or ‘financial constraints’ were often given as reasons. Some respondents mentioned that it was rather complex to perform a good assessment of a patient, especially if the older adult was not able to give all information themselves, and information needed to be gathered from other disciplines or informal caregivers. Lastly, some respondents stated that even though people responsible for nutritional care were able to see and/or screen all patients, there was no time left to support the delivery of nutritional care.

### 3.3. Screening for (Risk of) Malnutrition

The second part of the survey focused on the screening and treatment of patients with (risk of) malnutrition. Most respondents (73%) reported that all of their patients were screened for (risk of) malnutrition (Table 3). However, in Eastern countries, this percentage was only 28%, and the respondents from these countries mostly indicated only screening ‘selected patients’. When asking which ‘selected patients’ were screened, the following answer categories were given: (1) patients in a specific integrated care pathway or with a specific disease (e.g., COPD, stroke, amputation, anorexia, CVD); (2) if the physician gave advice to screen; (3) patients below a certain BMI (e.g., <22 or <20); (4) patients with dysphagia; (5) depended on the admitting nurse.

Screening was performed with a validated screening tool (e.g., MNA, MUST, SNAQ) for 95% of the respondents. The use of national and international guidelines was reported in 79% of the cases, with the highest rate in the Northern/Nordic countries (94%) and the lowest percentage in the Central/continental countries (74%). After screening, 42% of respondents indicated performing a more detailed nutritional assessment. Large differences could be seen in answers to these questions among the geographical areas, where 71% of respondents from Eastern countries indicated performing a more detailed nutritional screening, whilst this was only 19% in the Central/continental countries.

### 3.4. Treating People with (Risk of) Malnutrition

When looking at interventions used to treat people at risk of malnutrition, a treatment plan based on nutritional care guidelines was available according to 68% of respondents (Table 4). This treatment plan mostly consisted of dietary counseling (85%), followed by the prescription of oral nutritional supplements (ONSs) (76%) and dietary adaptations (75%). Respondents from the Central/continental countries mostly relied on dietary counseling (93%) whilst the prescription of ONSs was at 100% among respondents from Eastern countries. In the Northern and Southern countries, there was an equal distribution in the use of these three treatment options. No respondents indicated not treating people with a risk of malnutrition.

The table shows that slightly interventions were performed more often if patients were suffering from malnutrition compared to when patients had a risk of malnutrition; dietary counseling was reported by 90% of respondents for people with malnutrition compared to 85% for people with a risk of malnutrition. For the prescription of ONSs, this was 85% compared to 76%, and for dietary adaptations, this was 78% versus 75%. In the Eastern countries, patients with a risk of malnutrition mostly received oral nutritional supplements, while for patients with malnutrition, this was 57%.

### 3.5. Screening for and Treating Frailty and Sarcopenia

**Figure 2:**
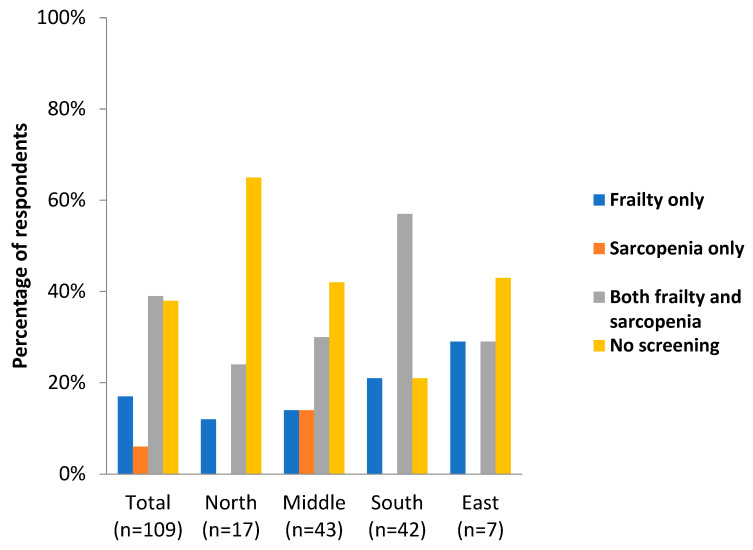
Screening for frailty and sarcopenia in different geographical areas in Europe.

The third part of the questionnaire focused on the screening and treatment of frailty and sarcopenia. The results showed that in 38% of the cases, the rehabilitation centers did not screen for frailty or sarcopenia; this trend was predominately seen in Nordic countries (65%) (Figure 2).

As shown in Table 5, physical exercise was reported as being used as a treatment strategy for sarcopenia by 85% of respondents, with the lowest rate in Northern countries (63%) and the highest rate in Eastern countries (100%). When looking at the other treatment options, food fortification and the prescription of (high-protein) oral nutritional supplements were used most often to treat sarcopenia, with some variations among countries.

When looking at the treatment options for frailty, similar rates to those for the treatment of sarcopenia were found. Oral nutritional supplements were more frequently reported as being used to treat sarcopenia (73%) compared to when treating frailty (66%). In addition, a higher number of respondents indicated that frailty was not being treated in their facility (11%) than for sarcopenia (8%). Treating sarcopenia and frailty was mostly done by performing physical exercise (87%) and food fortification (79%).

In general (>90%), when patients were diagnosed with sarcopenia or frailty, they were also screened and/or treated for malnutrition.

## 4. Discussion

This is the first study to examine nutrition practices in GR clinics across Europe. The primary aim was to obtain an overview of current care structures and resources for the diagnosis and treatment of malnutrition. Since the geriatric syndromes of sarcopenia and frailty are closely linked to malnutrition, the diagnostics and treatment of these syndromes were also examined. Even though the literature shows high prevalence rates and overlap of malnutrition, sarcopenia, and frailty in acute hospitals and geriatric rehabilitation [2,10,18,19,20,21,22], we found that patients were more often screened for malnutrition than for sarcopenia and frailty. For example, screening for sarcopenia and/or frailty in patients admitted to GR was reported in only 63% of the respondents. Inadequate screening for sarcopenia is a likely contributor to underdiagnosis [26]. Screening for malnutrition was only reported to be routinely carried out by 73% of the respondents.

Our study also shows some differences among geographical areas in Europe. For example, Central European countries seem to have less frequent thorough nutritional assessments than countries in the North, South, or East. Countries in the South of Europe are less likely to have dedicated nutritional teams, but are more likely to screen for sarcopenia and frailty. These differences might be explained by differences in structures and organization in geriatric rehabilitation [27]—particularly around the availability of expertise within teams. In parts of Europe, there is still room for improvement in nutritional screening and care.

The fact that more than half of all respondents reported staff resources to be a major constraint raises the question of whether the link between nutrition and (geriatric) rehabilitation outcomes—and those between nutrition, sarcopenia, and frailty—is sufficiently recognized in daily GR practice.

Although the majority of the professionals stated that basic nutritional care is in place (87% stated there is at least one individual responsible for nutrition and 57% reported a team focused on nutrition in the facility), more than half of the respondents stated that the responsible individual(s) and/or team members did not have enough time to support nutritional care. International and national guidelines for the diagnosis and treatment of malnutrition were not widely adhered to.

Previous studies have already shown a significant co-occurrence of malnutrition, sarcopenia, and frailty in older persons in various settings [21,29,30,31,32,33]—for example, in a GR setting based in Japan, the combined occurrence of sarcopenia and malnutrition was reported in 23.5% of patients [32]. The conditions of malnutrition, sarcopenia, and frailty overlap in etiologies, and symptoms and have similar consequences for older patients, including falls, fractures, hospitalization, morbidity, and mortality [5,29,34,35,36,37].

Robust evidence demonstrates the positive effect of nutritional and physical exercise interventions alone, as well as in combination. Effects have been shown on nutritional status, muscle mass, and function across various settings [9,38,39,40,41,42,43,44,45] This supports the case for early screening and diagnosis of malnutrition and sarcopenia, as well as proper treatment in the form of nutritional support and physical therapy/exercise in geriatric rehabilitation.

Despite the evidence around the positive impact of nutrition and exercise, nutritional education for healthcare professionals, including medical doctors and nurses, is still in its infancy [46,47]. Additionally, older people themselves are often unable to recognize changes in their own nutritional status, and they gravely underestimate their nutritional risk [48]. An increased focus on professional and public education is required.

### 4.1. Strengths and Limitations

This is the first study to provide insights into nutritional care practices in GR facilities across Europe; therefore, these first descriptions provide valuable insights into barriers and possibilities for improvement. Another strength of this study is the large variety of responding countries (n = 25), which provided a solid representation of European nutritional care practices in geriatric rehabilitation.

There are a number of limitations. First, some countries were overrepresented, with Spain (n = 19) and the Netherlands (n = 15) being the highest, compared to countries such as Latvia and Estonia, which had the lowest numbers of respondents (both n = 1). As a consequence, we did not perform any statistical tests or put firm conclusions on comparisons between countries and regions. Second, due to differences in nutritional care structures in GR facilities across Europe, we used rather broad inclusion criteria for the participants. We included any professionals responsible for or knowledgeable about nutritional care practices in geriatric rehabilitation. As a consequence, the background characteristics of participants included their roles (dietitians, nutritionists, geriatricians, etc.) and years of working experience. Due to the professional selection of the respondents, we assume that biases are likely, though it is difficult to be sure of the way in which they will influence the findings. Our work is predominantly hypothesis-generating, and any such biases are, therefore, unlikely to diminish the importance of the findings.

### 4.2. Implications for Practice and Research

Our study showed that across Europe, there is still room for improvement in the structures and processes of nutritional care in geriatric rehabilitation. First, as many respondents reported staff resource constraints, a renewed focus on recruiting and training in nutritional expertise as part of GR workforce strategy is required. Day-to-day care practices need modification to include the full nutritional care cycle of nutritional screening, assessment, treatment, and follow-up. As countries can learn from each other, we recommend the development of an infrastructure for describing and sharing the best practices in nutritional care in geriatric rehabilitation, including regular benchmarking of outcomes. Developments and support needs in individual regions of Europe can then be identified and addressed in a more evidence-based way.

Because malnutrition, sarcopenia, and frailty co-present and are intertwined in patients admitted to GR facilities, work is needed to develop an integrated approach to the screening and treatment of all three. This will require further collaboration between international research societies, such as the EUGMS and ESPEN, for the adaptation of current specific guidelines focusing on the diagnosis and treatment of malnutrition, sarcopenia, and frailty as isolated syndromes.

## 5. Conclusions

This study shows variations in nutritional care in geriatric rehabilitation across Europe. These differences can likely be explained by differences in the structures and organization of care, as well as differences in focuses related to malnutrition, frailty, and sarcopenia. In general, the importance of adequate nutritional care was underlined by the respondents, but staff resource constraints were a universal feature. Given the inter-related nature of frailty, sarcopenia, and malnutrition, a structured and integrated approach to screening for and treating all three, e.g., in guidelines, should be developed.

## Figures and Tables

**Table 1:** Background characteristics of the participants.

Profession	Total (n = 109)	North (n = 17)	Central (n = 43)	South (n = 42)	East (n = 7)
Geriatrician	50%	24%	44%	64%	57%
Dietician	37%	65%	44%	21%	14%
Physiotherapist	1%	0%	0%	2%	0%
Other	13%	12%	12%	12%	29%
Work experience in profession (years)					
0–5	19%	35%	9%	20%	43%
6–10	18%	18%	16%	17%	29%
11–15	18%	12%	16%	22%	14%
16–20	12%	0%	14%	15%	14%
>20	33%	35%	44%	27%	0%
Followed education on nutrition					
	39%	41%	30%	45%	43%

**Table 2:** Dedicated nutritional care in GR centers.

	Total (n = 109)	North (n = 17)	Central (n = 43)	South (n = 42)	East (n = 7)
Is there an individual dedicated to nutritional care?					
Yes	87%	94%	98%	76%	71%
Profession of individual dedicated to nutritional care					
Dietician	67%	94%	88%	41%	29%
Nutritionist	19%	18%	5%	33%	29%
Geriatrician	6%	0%	5%	12%	0%
Dietetic assistant	6%	0%	12%	2%	0%
Other	11%	12%	7%	12%	29%
Nutritional team in GR center					
Yes	57%	53%	58%	25%	71%
Members of the nutritional team					
Dietician	56%	53%	58%	55%	57%
Speech therapist	37%	41%	44%	21%	71%
Occupational therapist	23%	24%	26%	17%	43%
Dentist	6%	12%	9%	0%	0%
Other (physician, nurses, pharmacist)	24%	23%	5%	29%	17%
Do you think the individual/team has enough time to dedicate to the patients?					
Yes	56%	44%	67%	47%	67%

**Table 3:** Screening for (risk of) malnutrition.

	Total (n = 109)	North (n = 17)	Central (n = 43)	South (n = 42)	East (n = 7)
Do you screen for (risk of) malnutrition?					
Yes, all patients	73%	77%	77%	76%	29%
Yes, but only selected patients	24%	12%	21%	24%	71%
No	3%	12%	2%	0%	0%
Do you use (inter)national nutritional screening guidelines?					
Yes	79%	94%	74%	76%	86%
How do you screen for (risk of) malnutrition?					
Standard screening tool (e.g., MUST, MNA, SNAQ)	95%	100%	91%	98%	100%
BMI	60%	65%	61%	57%	57%
Weight	61%	59%	63%	62%	43%
Clinical presentation	60%	47%	58%	67%	57%
Other (mostly albumin, biomarkers)	17%	6%	19%	17%	28%
Do you perform a more detailed nutritional assessment?					
Yes	42%	59%	19%	55%	71%

**Table 4:** Treating people with (risk of) malnutrition.

	Total (n = 109)	North (n = 17)	Central (n = 43)	South (n = 42)	East (n = 7)
Treatment plan for people at risk of malnutrition based on nutritional care guidelines?					
Yes	68%	52%	72%	69%	71%
No	19%	29%	12%	21%	29%
Not applicable	13%	18%	16%	10%	0%
What does the treatment plan for patients at risk for malnutrition consist of?					
Dietary counseling	85%	94%	93%	74%	86%
Prescription of ONS	76%	94%	65%	76%	100%
Dietary adaptations	75%	94%	70%	74%	71%
Other	7%	6%	7%	7%	14%
No treatment	0%	0%	0%	0%	0%
What does the treatment plan for patients with malnutrition consist of?					
Dietary counseling	90%	94%	95%	83%	86%
Prescription of ONS	85%	100%	84%	86%	57%
Dietary adaptations	78%	88%	72%	79%	86%
No treatment	1%	0%	2%	0%	0%

**Table 5:** Treatment of sarcopenia and frailty.

SARCOPENIA	Total (n = 106)	North (n = 16)	Central (n = 42)	South (n = 40)	East (n = 7)
How do you treat sarcopenia?					
Physical exercise (aerobic/resistance/balance)	85%	63%	91%	85%	100%
Food fortification/high-energy and/or high-protein diet/additional food	81%	63%	86%	80%	100%
Texture-modified food	39%	19%	36%	48%	57%
ONSs	73%	63%	71%	80%	71%
Other diet modifications	35%	25%	41%	33%	43%
Sarcopenia is not being treated	8%	25%	2%	5%	14%
If a patient is diagnosed with sarcopenia, do you also screen for and/or treat malnutrition?					
Yes	92%	86%	93%	95%	86%
**FRAILTY**	**Total (n = 103)**	**North (n = 16)**	**Middle (n = 39)**	**South (n = 41)**	**East (n = 7)**
How do you treat frailty?					
Physical exercise (aerobic/resistance/balance)	87%	69%	87%	93%	100%
Food fortification/high-energy and/or high-protein diet/additional food	79%	69%	74%	85%	86%
Texture-modified food	48%	50%	33%	56%	71%
ONSs	66%	69%	64%	68%	57%
Other diet modifications	41%	38%	41%	39%	57%
Frailty is not being treated	11%	19%	13%	7%	0.0%
If a patient is diagnosed with frailty, do you also screen for and/or treat malnutrition?					
Yes	90%	80%	93%	93%	86%

## Data Availability

Data are available from the corresponding author upon request.

## Supplementary Materials

The following supporting information can be downloaded at: https://www.mdpi.com/article/10.3390/jcm12082918/s1, Survey nutritional care in geriatric rehabilitation.
