# Supplementary material for: Nutritional Care Practices in Geriatric Rehabilitation Facilities across Europe: A Cross-Sectional Study
Source: J Clin Med. 2023 Apr 17;12(8):2918. doi: 10.3390/jcm12082918 (PMC10142565; doi:10.3390/jcm12082918)
Supplement: Supplementary file 1 [file jcm-12-02918-s001.zip › jcm-2201794-supplementary.pdf]

2021 EuGMS SIG Questionnaire: Nutritional care in geriatric rehabilitation

Thank you for participating in this questionnaire. This questionnaire is developed by the EuGMS Special Interest Group on Geriatric Rehabilitation.

We acknowledge that Europe is currently in the midst of the COVID-19 pandemic, which heavily affects the health care systems. As health care professionals you are under massive amounts of pressure and your time is very valuable. We therefore are very appreciative of your participation.

With this questionnaire we aim to gain more insights into the current practices in nutritional care in geriatric rehabilitation, which is also very important for the recovery of COVID-19 survivors.

We ask you to complete this questionnaire and provide us with your expertise, preferably through the person responsible for nutrition care at your rehabilitation center. We aim to conclude this questionnaire halfway 2021, to bring our results into the current course of treatment from GR services as soon as possible.

If you have any questions or remarks, do not hesitate to contact us via
stefan.grund@bethanien-heidelberg.de

Thank you for your participation!

* Required

General background questions

1. What is your profession? *

Mark only one oval.

- ◯ Geriatrician
- ◯ Physiotherapist
- ◯ Dietician
- ◯ Other: ____________________

2. How many years have you worked in this profession?

3. In which country do you work? *

4. In which country did you follow your educational training? *

5. Did you follow a specific nutritional training related to rehabilitation or geriatric rehabilitation? *

Mark only one oval.

- Yes
- No

6. Can you describe the geriatric rehabilitation center in which you work? (Number of beds, specialization, located in a hospital or not, etc.) *

Skip to section 3 (Part I: General nutritional care in your geriatric rehabilitation center)

Part I: General nutritional care in your geriatric rehabilitation center

Nutritional Care

7. 1a. Is there a person dedicated to nutrition care available in your geriatric rehabilitation center? *

Mark only one oval.

- ◯ Yes
- ◯ No

8. 1b. If yes, who? (Please select all that apply)

Check all that apply.

- ☐ Dietician
- ☐ Nutritionist
- ☐ Dietetic assistant
- Other: ☐ ____________

9. 2a. Is there a nutritional team in your geriatric rehabilitation center? *

Mark only one oval.

- ◯ Yes
- ◯ No

10. 2b. If yes, who is part of this team? (tick all that apply)

Check all that apply.

- ☐ Dietician
- ☐ Speech therapist
- ☐ Occupational therapist
- ☐ Dentist
- Other: ☐ ____________

11. 3. How much time does the dedicated person or nutritional team have per patient? (for example; 30 minutes, divided over 3 sessions, or: 20 minutes, only at admission)

12. 4a. Do you think this person or this team has enough time to dedicate to the patients?

Mark only one oval.

- Yes
- No (please go to question 4b)

13. 4b. If you think this person or this team doesn't have enough time, why do you think this is?

Screening for Malnutrition

14. 5a. Do you screen your patients for malnutrition/risk of malnutrition? *

Mark only one oval.

- No (please go to question 6a)
- Yes, but only selected patients: based on physical manifestations of malnutrition and/or diagnosis and comorbidities at admission (please go to question 6b)
- Yes, everyone (please go to question 7)

15. 6a. If you don’t screen for malnutrition, why not? (Please select all that apply)

Check all that apply.

- [ ] Lack of resources
- [ ] Lack of expertise
- Other: [ ] ____________________

16. 6b. If you only screen selected patients, please elaborate on how you do this (for example: only patients with heart failure, only patients with BMI<20, etc):

____
____
____
____
____

17. 7a. Are there (inter)national guidelines on nutritional screening that you implement into your practice?

Mark only one oval.

- ( ) No
- ( ) Yes (please go to question 7b)

18. 7b. Please indicate which guideline(s) you use for nutritional screening:

____
____
____
____
____

19. 8a. How do you screen for malnutrition/risk of malnutrition? (Please select all that apply)

Check all that apply.

- [ ] Standard screening tool (MUST, MNA, SNAQ, …)
- [ ] BMI
- [ ] Weight
- [ ] Clinical presentation

Other: [ ] ______________________

20. 8b. If you use a screening tool, which screening tool do you use? (Please select all that apply)

Check all that apply.

- [ ] MNA
- [ ] MNA-SF
- [ ] MST
- [ ] MUST
- [ ] SNAQ

Other: [ ] ______________________

21. 9a. In addition to screening for malnutrition, do you also perform a more detailed nutritional assessment? (for example GLIM or ESPEN assessment)

Mark only one oval.

- ( ) Yes (please go to question 9b)
- ( ) No

22. 9b. If yes, which assessment? (Please select all that apply)

Check all that apply.

- [ ] GLIM (Cederholm et al., J Cachexia Sarcopenia Muscle 2019)
- [ ] ESPEN (Cederholm et al., Clin Nutr 2015)

Other: [ ] ______________________

Nutritional treatment

23. 10a. Is the treatment plan for patients at risk for malnutrition, and patients diagnosed with malnutrition, based on nutritional care guidelines? *

Mark only one oval.

- Yes (please go to question 10b)
- No
- Not applicable

24. 10b. Please indicate which guideline(s) you use for the treatment plan:

____
____
____
____
____

25. 11. What is the treatment for patients at risk for malnutrition? (Please select all that apply)

Check all that apply.

- [ ] No treatment
- [ ] Dietary counselling
- [ ] Prescription of oral nutritional supplements (ONS)
- [ ] Implement diet adaptations (if yes please go to question 12)

Other: [ ] ______________________

26. 12. Which dietary changes do you implement? (Please select all that apply)

Check all that apply.

- [ ] Texture adapted diet
- [ ] Increased calories
- [ ] Increased protein
- [ ] Increased fruit and vegetables
- [ ] Increased fibers
- [ ] Increased variety

Other: [ ] ______________________

27. 13. What is the treatment for patients with malnutrition? (Please select all that apply)

Check all that apply.

- [ ] No treatment
- [ ] Dietary counselling
- [ ] Implement diet adaptations (if yes please go to question 14)
- [ ] Prescription of Oral nutritional supplements (ONS)

28. 14. Which dietary changes do you implement? (Please select all that apply)

Check all that apply.

- [ ] Texture adapted diet
- [ ] Increased calories
- [ ] Increased protein
- [ ] Increased fruit and vegetables
- [ ] Increased fibers
- [ ] Increased variety

Other: [ ] ______________________

29. 15a. If you prescribe oral nutritional supplements (ONS), for what duration do you typically prescribe the ONS and at what dosage do you prescribe?

30. 15b. If you prescribe ONS, which products do you use, and why?

31. 16a. For patients without risk of malnutrition and malnutrition, do you have a standard nutritional care plan?

Mark only one oval.

- Yes- please go to 16b.
- No

32. 16b. If yes, please describe what the standard nutritional care plan entails:

33. 17. If a patient is diagnosed with malnutrition, do you also screen and/or treat for sarcopenia and/or frailty?

Mark only one oval.

- Yes
- No

Part II: Sarcopenia and Frailty

Screening

34. 18. Do you screen patients in your rehabilitation center for sarcopenia and/or frailty? *

Mark only one oval.

- Yes: for frailty only (please go to question 19)
- Yes: for sarcopenia only (please go to question 19)
- Yes: for both frailty and sarcopenia (please go to question 21)
- No: I don't screen for sarcopenia or frailty (please go to question 20):

35. 19. If you screen for either sarcopenia or frailty, and not both, it is because (please select all that apply):

Mark only one oval.

- Lack of expertise on the other
- Lack of resources for the other
- Lack of time to screen for both
- Don't see the value of screening for both
- Other: ______________________

36. 20. If you don't screen for either sarcopenia or frailty, it is because:

Mark only one oval.

- Lack of resources
- Lack of expertise
- Other: ____________________

Treatment of sarcopenia and frailty

37. 21. How do you treat sarcopenia once diagnosed? (Please select all that apply)

Check all that apply.

- [ ] Physical Exercise: Aerobic
- [ ] Physical Exercise: Resistance
- [ ] Physical Exercise: Balance
- [ ] Nutritional intervention/ Diet modification: Food fortification/ high energy and/or high protein diet/ additional food
- [ ] Nutritional intervention/ Diet modification: textured modified diet
- [ ] Nutritional intervention/ Diet modification: Oral nutritional supplements (ONS)
- [ ] Nutritional intervention/ Diet modification: other diet modification
- [ ] I don't treat sarcopenia

Other: [ ] ____________________

38. 22. How do you treat frailty once diagnosed? (Please select all that apply)

Check all that apply.

- [ ] Physical Exercise: Aerobic
- [ ] Physical Exercise: Resistance
- [ ] Physical Exercise: Balance
- [ ] Nutritional intervention/ Diet modification: Food fortification/ high energy and/or high protein diet/ additional food
- [ ] Nutritional intervention/ Diet modification: textured modified diet
- [ ] Nutritional intervention/ Diet modification: Oral nutritional supplements (ONS)
- [ ] Nutritional intervention/ Diet modification: other diet modification
- [ ] I don't treat frailty

Other: [ ] ____________________

39. 23a. If a patient is diagnosed with sarcopenia, do you also screen and/or treat for malnutrition?

Mark only one oval.

- Yes
- No

40. 23b. If a patient is diagnosed with frailty, do you also screen and/or treat for malnutrition?

Mark only one oval.

- Yes
- No

Part III: Barriers and enablers for prescribing oral nutritional supplements

41. 24. What are the barriers for prescribing oral nutritional supplements? (for example; availability of products, little knowledge of products, ...) *

42. 25. What are the enablers for prescribing oral nutritional supplements? (for example; clinical efficacy, positive feedback from patients, ...) *

43. 26. Do you follow-up with patients after prescribing oral nutritional supplements? *

Mark only one oval.

- Yes (please go to question 27)
- Yes, but not always (please go to question 27 & 28)
- No (please go to question 28)
- Other: _______________

44. 27. How much time usually passes before the follow-up appointment occurs?

Mark only one oval.

- Less than 2 weeks
- Less than one month
- Between 1 and 3 months
- Longer than 3 months
- Other: _______________

45. 28. What prevents you from following-up with a patient? (for example; lack of time, does not prescribe oral nutritional supplements, ...):

____

____

____

____

____

Part IV: Impact of nutritional interventions on various outcomes for patients with malnutrition and risk of malnutrition

29. How would you rate the impact of nutritional interventions using oral nutritional supplements on the following possible outcomes?

As “low impact”: score 1-3
As “impactful but not critical”: score 4-6
As “high impact”: score 7-9

46. Mortality *

Mark only one oval.

	1	2	3	4	5	6	7	8	9	
low impact	◯	◯	◯	◯	◯	◯	◯	◯	◯	high impact

47. Morbidity (hospital complications, infection, pressure ulcers) *

Mark only one oval.

	1	2	3	4	5	6	7	8	9	
low impact	◯	◯	◯	◯	◯	◯	◯	◯	◯	high impact

48. Functional status (mobility, ADL, physical performance) *

Mark only one oval.

	1	2	3	4	5	6	7	8	9	
low impact	◯	◯	◯	◯	◯	◯	◯	◯	◯	high impact

49. Cognitive status (including dementia and delirium) *

Mark only one oval.

	1	2	3	4	5	6	7	8	9	
low impact	◯	◯	◯	◯	◯	◯	◯	◯	◯	high impact

50. Nutritional status (weight change, BMI, skin folds, muscle mass) *

Mark only one oval.

	1	2	3	4	5	6	7	8	9	
low impact	⬭	⬭	⬭	⬭	⬭	⬭	⬭	⬭	⬭	high impact

51. Changes in dietary intake *

Mark only one oval.

	1	2	3	4	5	6	7	8	9	
low impact	⬭	⬭	⬭	⬭	⬭	⬭	⬭	⬭	⬭	high impact

52. Blood biomarkers (albumin, transferrin) *

Mark only one oval.

	1	2	3	4	5	6	7	8	9	
low impact	⬭	⬭	⬭	⬭	⬭	⬭	⬭	⬭	⬭	high impact

53. Falls *

Mark only one oval.

	1	2	3	4	5	6	7	8	9	
low impact	⬭	⬭	⬭	⬭	⬭	⬭	⬭	⬭	⬭	high impact

54. Quality of Life *

Mark only one oval.

	1	2	3	4	5	6	7	8	9	
low impact	◯	◯	◯	◯	◯	◯	◯	◯	◯	high impact

55. Frailty status *

Mark only one oval.

	1	2	3	4	5	6	7	8	9	
low impact	◯	◯	◯	◯	◯	◯	◯	◯	◯	high impact

56. Adverse events (diarrhea, nausea) *

Mark only one oval.

	1	2	3	4	5	6	7	8	9	
low impact	◯	◯	◯	◯	◯	◯	◯	◯	◯	high impact

Part V: Barriers and Enablers for adherence to nutritional treatment, in particular oral nutritional supplements

57. 30. What is the % of patients you would expect to fully adhere (takes oral nutritional supplements according to prescription >75% of the time) to their oral nutritional supplements prescription in geriatric rehabilitation? *

58. 31. What do you see as the three most important barriers to adherence to oral nutritional supplements, during geriatric rehabilitation? *

59. 32a. Between liquid and powder oral nutritional supplements, which format of oral nutritional supplements do you believe would have greater adherence for patients in rehab setting? *

Mark only one oval.

- Liquid
- Powder
- No difference between formats

60. 32b. If you think there is a difference between the formats- please explain why you think that is?

Other remarks

61. Do you have any other remarks on the nutritional care in your rehabilitation center?

Thank you for your cooperation!

This content is neither created nor endorsed by Google.

Google Forms
